# Using theatre to transform understanding and attitudes about asthma in African adolescents

**DOI:** 10.1093/heapro/daaf189

**Published:** 2025-11-19

**Authors:** Gioia Mosler, Lovemore Mzati Nkhalamba, Elizabeth Mkutumula, Emmanuel Addo-Yobo, Olayinka Olufunke Adeyeye, Bernard Arhin, Tunde Euba, Jeremy James, Stella Maleni, Refiloe Masekela, Reratilwe Mphahlele, Hilda Angela Mujuru, Sofia Muyemayema, Rebecca Nantanda, Olufemi Tunde Ojo, Sandra Kwarteng Owusu, Liz Steed, Ismail Ticklay, Lindsay Zurba, Jonathan Grigg

**Affiliations:** Centre for Genomics and Child Health, Blizard Institute, Faculty of Medicine and Dentistry, Queen Mary University of London, 4 Newark St, London E1 2AT, United Kingdom; Malawi Liverpool Wellcome Programme, P.O. Box 30096, Blantyre, Malawi; Malawi Liverpool Wellcome Programme, P.O. Box 30096, Blantyre, Malawi; Department of Child Health, School of Medical Sciences , Kwame Nkrumah University of Science and Technology (KNUST), Private Mail Bag, KNUST Kumasi, Ghana; Department of Medicine, Lagos State University College of Medicine, P.M.B. 21266., 1-5 Oba Akinjobi Way, G.R.A., Ikeja Lagos, Nigeria; Komfo Anokye Teaching Hospital, Okomfo Anokye Road, Kumasi, Ghana; Freelance Writer and Playwright, London, United Kingdom; Artistic Director of Tramshed, 51-53 Woolwich New Road, London SE18 6ES, United Kingdom, now Freelance; Makerere University Lung Institute, College of Health Sciences, Makerere University, 7s Upper Mulago Hill Rd, Kampala, Uganda; Department of Paediatrics and Child Health, Nelson R Mandela School of Clinical Medicine, College of Health Sciences, University of KwaZulu Natal, 719 Umbilo Road, Congella, Durban 4001, South Africa; Department of Paediatrics and Child Health, Nelson R Mandela School of Clinical Medicine, College of Health Sciences, University of KwaZulu Natal, 719 Umbilo Road, Congella, Durban 4001, South Africa; University of Zimbabwe Faculty of Medicine and Health Sciences, Department of Child, Adolescent and Women Health, Box A178, Avondale, Harare, Zimbabwe; University of Zimbabwe Faculty of Medicine and Health Sciences, Department of Child, Adolescent and Women Health, Box A178, Avondale, Harare, Zimbabwe; Makerere University Lung Institute, College of Health Sciences, Makerere University, 7s Upper Mulago Hill Rd, Kampala, Uganda; Department of Medicine, Lagos State University College of Medicine, P.M.B. 21266., 1-5 Oba Akinjobi Way, G.R.A., Ikeja Lagos, Nigeria; Department of Child Health, School of Medical Sciences , Kwame Nkrumah University of Science and Technology (KNUST), Private Mail Bag, KNUST Kumasi, Ghana; Wolfson Institute of Population Health, Barts and the London School of Medicine and Dentistry, Queen Mary University of London, 58 Turner Street, London E1 2AB, United Kingdom; University of Zimbabwe Faculty of Medicine and Health Sciences, Department of Child, Adolescent and Women Health, Box A178, Avondale, Harare, Zimbabwe; Education for Health Africa, Kingfisher Office Park, 28 Siphosetu Rd, Mount Edgecombe, Durban 4301, South Africa; Centre for Genomics and Child Health, Blizard Institute, Faculty of Medicine and Dentistry, Queen Mary University of London, 4 Newark St, London E1 2AT, United Kingdom

**Keywords:** educational theatre, asthma, adolescents, school, Africa

## Abstract

Asthma is a common non-communicable disease in children and adolescents. In the Global South, many children are either undiagnosed or under-treated. Health education through theatre has emerged as an innovative and effective method for disseminating health-related information in Africa for conditions such as HIV/AIDS. In this study, we therefore aimed to assess the impact of an asthma awareness theatre, which was adapted from a UK-based play by local theatre groups in six sub-Saharan African countries. This theatre aimed to improve asthma knowledge, and attitudes, dispel myths, and reduce stigma around asthma among adolescents. The study employed a pre-post design, using questionnaires to assess changes in asthma knowledge before and after the theatre, testing for statistically significant changes using Wilcoxon Signed Rank Tests. Adolescents (11–17 years) in Ghana, Malawi, Nigeria, South Africa, Uganda, and Zimbabwe were recruited (*n* = 3534) through local schools. Nearly all participants stated that the performance was fun (90.7%) and informative (98.1%). Asthma knowledge significantly improved (*P* < 0.01), including the belief that asthma is not contagious, which rose from 59.7% before to 78.3% after the theatre. To improve health-seeking behaviour and address under-diagnosis and undertreatment of asthma in sub-Saharan African societies, educating young people about asthma and reducing stigma around the condition is of particular importance. We conclude that theatre is an effective way to educate adolescents about asthma at scale across societies in Africa.

Contribution to Health Promotion

- It is both feasible and acceptable to run educational theatre about asthma in schools in six African countries.
- Educational theatre can significantly improve asthma knowledge and attitudes, as well as dispel myths about asthma in African adolescents.
- The asthma theatre was acceptable to participants, and perceived to be entertaining and educational across all six African sites, proving the success of involving local theatre groups for the cultural adaptation of the script to the local context.

## BACKGROUND

Asthma is one of the most prevalent non-communicable diseases globally, affecting around 300 million people, with the highest mortality rates occurring in low- and middle-income countries (Global Initiative for Asthma 2024).

In a study of 27 272 adolescents across six African cities we previously found that one in eight participants experienced asthma symptoms (Oyenuga *et al.* 2024). Despite this, only 19.9% (644/3236) of participants with symptoms of asthma had a formal diagnosis of asthma. Even among those with a formal diagnosis of asthma, most did not regularly use asthma medication (Oyenuga *et al.* 2024). A general lack of awareness of the burden of asthma in Africa was highlighted by Masekela *et al* (Masekela *et al.* 2022), with poor symptom recognition both by patients and health care workers. One cross-sectional study of 519 adult patients with chronic respiratory symptoms in three African countries showed evidence of under-reporting of asthma by patients, with 29% of those with a clinician diagnosis of asthma denying they had asthma, while they also found evidence of possible misclassification of COPD as asthma of up to 39% of cases, as well as evidence of the reverse by 18% −21% of patients (Binegdie *et al.* 2022). In addition, cultural misconceptions, such as the belief that asthma is contagious or that it can be cured were evident from focus group discussions, which further hinder effective disease management (Naidoo *et al.* 2023a, 2023b). Stigma associated with asthma also contributes to delays in families seeking treatment and reluctance to adhere to prescribed therapies, which can result in exacerbations and complications (Lenney *et al.* 2018).

Theatre has been used as an effective and engaging strategy for the dissemination of health information in Africa, especially to educate about HIV/AIDS, and in malaria prevention. A meta-analysis of educational theatre to improve HIV/AIDS-related knowledge showed significantly higher odds among the intervention groups of correct understanding of several knowledge endpoints, such as risk reduction through condom use (Faust and Yaya 2018). A systematic review of educational interventions about malaria in sub-Saharan Africa showed that art-focused community-based educational approaches, such as drama, were effective to improve malaria knowledge (Owusu-Addo and Owusu-Addo 2014), with one of the studies successfully involving schoolchildren through drama (Ayi *et al.* 2010). Further evidence that health promotion through theatre works for children comes from a systematic review of school-based drama interventions for health promotion. The review included nine relevant studies, most of which succeeded in the short-term in increasing knowledge and positive attitudes related to health behaviour among schoolchildren (Joronen *et al.* 2008). Theatre engages audiences, both emotionally and intellectually, making it an effective tool for public health messaging (Mbizvo 2006, WHO Regional Office for Europe 2019). Furthermore, several studies indicated that educational theatre about health-related topics can reduce stigma, for example, regarding mental illness (Lee *et al.* 2022), or in relation to HIV/AIDS prevention (Logie *et al.* 2019).

Based on the above evidence, we wanted to explore the following research question: Can educational theatre improve health perceptions about asthma in African adolescents? We therefore designed a theatre aimed to improve asthma knowledge and attitudes towards asthma, dispel myths, and reduce stigma around asthma among adolescent schoolchildren. Our primary objective was to assess how effective this theatre is at increasing asthma-related knowledge and change attitudes about the disease among participants. The secondary objective was to understand acceptability of the performance by the participating adolescents. We report the results of the evaluation completed by adolescent students both with and without asthma symptoms.

## METHODS

### Study design

This study was part of the larger Achieving Control of Asthma in Children in Africa (ACACIA) study, which aimed to understand and improve asthma in African adolescents. Several aspects of the ACACIA study have previously been published, including the study protocol (Mosler *et al.* 2020), a paper on asthma symptoms, severity and control in African adolescents (Oyenuga *et al.* 2024), a paper on air pollution exposures of African adolescents with asthma symptoms (Lim *et al.* 2024), and two papers on barriers to children’s asthma care in Africa (Naidoo *et al.* 2023a, 2023b). These previous publications focused on describing the situation of adolescents with asthma in Africa. For this study, we used a pre-post design to evaluate an educational theatre about asthma across six African cities: Kumasi, Ghana; Blantyre, Malawi; Lagos, Nigeria; Durban, South Africa; Kampala, Uganda; and Harare, Zimbabwe.

### The educational theatre

The ‘In Control—Africa’ theatre is a live performance followed by an interactive workshop, designed to educate adolescents about asthma, and influence their attitudes towards the condition. Leventhal’s theory of self-regulation underlies the messages conveyed by the theatre, with a focus of noticing the problem and trying to make sense of it, and with the goal to empower young people about their health, their condition, and their symptoms (Leventhal *et al.* 1998). The educational theatre was originally developed for UK secondary schools by writer Tunde Euba as part of a collaboration between Queen Mary University of London and a specialist Theatre in Education Company and Youth Theatre, called the Greenwich & Lewisham Young People’s Theatre (GLYPT) in London—subsequently renamed as Tramshed (Mosler and Euba 2018). It was then adapted as ‘In Control—Africa’ to the cultural context of each sub-Saharan African country. To inform the African adaptation, the writer spent a week in Nigeria and Malawi, running workshops with the research team including healthcare workers, as well as with young asthma sufferers and in schools, exploring the specific country issues around asthma on the ground for these young people. The Nigerian-born writer then developed an initial version of the play from a Nigerian cultural experience and perspective (script available from corresponding author upon request). This Nigerian version of the play was then provided to theatre companies and local ACACIA research teams in each of the other five countries for local adjustments in order to make the characters, names, and other cultural specifics appropriate for each of those countries. The theatre professionals in those countries adapted the language, e.g. using local idioms, place names, celebrity references, nicknames, and the tone; whilst research teams were actively involved throughout the process to ensure that the text remained true to the objectives and educational values.

The script was originally written in English in all six African countries. The research teams in Malawi, Uganda, and South Africa furthermore translated the script into local languages (Chichewa, Luganda, and IsiZulu, respectively). The local theatre teams then performed the play and workshop. The performances were arranged and supervised by the research teams, who in turn were supported and mentored by the London-based theatre team. The storyline revolved around Jai, a teenage girl with asthma who loves playing football but struggles to manage her symptoms. Through interactions with her family, friends, and healthcare provider, Jai learns about the importance of using her inhaler, avoiding triggers, and seeking support from others. The theatre addresses common misconceptions about asthma, such as the belief that it is contagious, while emphasizing the emotional and social aspects of living with a chronic illness. Following the performance, an interactive workshop was held in which actors remained in character to engage with the audience. This provided an opportunity for adolescents to ask questions, discuss issues raised in the play and suggest potential solutions for Jai’s challenges. The interactive nature of the theatre allowed participants to relate the lessons from the play to their own experiences, reinforcing key health messages, such as the need for medication in asthma management.

### Study population and setting

The theatre was delivered in six sub-Saharan African cities representing diverse cultural and linguistic contexts in the region. Videos from some of the performances can be found here: https://www.acacia-asthma.org/resources. Target participants were adolescents, aged between 11 and 17, from ACACIA study schools. The schools’ management consented to take part in the study. As the theatre is delivered to whole classes or year groups—and not selected individuals—it was considered sufficient to seek head-teacher consent, as parent/carer consent in this situation has the risk to reduce response rates and introduce selection biases (Bonell *et al.* 2023). Schools were responsible to inform parents and students about the theatre ahead of time, and students had the opportunity to opt out of attending the performance with their teachers. Schools were sensitised to asthma-related research, as they had previously received visits from our research teams to collect data on asthma symptoms (Oyenuga *et al.* 2024). Performances for relevant year groups were arranged in conversation with the school. Some of our sites arranged additional community performances for a wider audience, which included school staff, parents, and members of the local community. For the results presented here, we focus on adolescents who attended the theatre.

### Data collection and analysis

Educational theatre and data collection took part between March and December 2021 in urban and suburban African schools. A pre- and post-theatre questionnaire assessed changes in knowledge and attitudes. It was based on items from the original UK-based theatre study, (Harris *et al.* 2022) with input from all African research teams (questionnaire available from corresponding author upon request). While the questionnaire was not formally validated, it had face validity, as it was tested by local teams for clarity and cultural appropriateness.

Participants completed a questionnaire before the theatre to assess their baseline knowledge of asthma, including beliefs about asthma transmission, chronicity, and the emotional and physical impact of the disease. Participants completed the same questionnaire to assess changes in their knowledge and attitudes towards asthma, after watching the play and participating in an interactive workshop. Additional questions were added in the post-theatre questionnaire to gauge participants’ acceptability and enjoyment of the play and their perceived impact on how they understand asthma. Answer formats were Yes/No, or a five-point Likert scale.

The questionnaires were available in local languages, and research assistants were on hand to provide clarification or assistance as needed. The questionnaires were completed on paper and then transcribed into REDCap by each local research team. Data cleaning and collation were undertaken centrally and included frequent communication with the site teams who had collected the data (e.g. to clarify reasons for missing data). This process faced several months of delays due to multiple personnel changes and key people leaving towards the end of the project.

Results are presented by country and for all participants. For yes/no answer options to knowledge questions, answers are summarized as percentage of correct answers compared with all answers. If responses were collected as a 5-point Likert scale, answers are presented as median, interquartile range, and as percentage of affirmative answers (agree or strongly agree; a little or a lot) compared to all answers. To test if there was a significant change in correct or affirmative responses from before to after the theatre performance, Wilcoxon signed-rank tests were applied to compare the proportion of correct to non-correct, or affirmative to non-affirmative answers between before and after the theatre. A sensitivity analysis was carried out to assess if there is a significant difference between male/female responses (see Supplement). Calculations were made using IBM SPSS, version 28.

### Ethical approvals

The ACACIA study was reviewed by the Queen Mary Ethics of Research Committee in the UK (Reference: QMERC2018/61). In addition, each collaborating African centre received local ethical approval for the study as follows: Malawi: KUHeS College of Medicine Research Ethics Committee, (Reference: P.10/18/2494). Nigeria: Lagos State/Lagos State University Teaching Hospital Health Research and Ethics Committee (References: NHREC04/04/2008; LREC 06/10/1084). Uganda: Mulago Hospital Research Ethics Committee (Reference: MHREC 1514); and National Council for Science and Technology (Reference: SS 4940). South Africa: Biomedical Research Ethics Committee of University of KwaZulu Natal (Reference: BF002/19) and Department of Basic Education KwaZulu Natal and Department of Health KwaZulu Natal (Reference: KZ_201902_037). Zimbabwe: The City Health Ethics Board, Joint Research Ethics Committee, the Medical Research Council of Zimbabwe and the Research Council of Zimbabwe (MRCZ/A/2415). Ghana: Committee on Human Research, Publication and Ethics a joint committee of the School of Medical Science, Kwame Nkrumah University of Science and Technology and the Komfo Anokye Teaching Hospital, which is registered with the US Department of Health and Human Services, Office for Human Research Protection (Reference: CHRPE/AP/074/19). All the above ethics committees approved the conduct of theatre performances as part of the wider ACACIA study without specific individual level or parental consent for theatre audiences. School-level consent was provided for the ACACIA study, including the theatre, from participating schools. Detailed and approved consent procedures were in place for other parts of the ACACIA study for adolescents who took part in individual data collection about respiratory symptoms, or lung function testing (Oyenuga *et al.* 2024).

## RESULTS

The teams visited 38 schools to conduct 53 performances with school student audiences, plus 15 performances including the wider community, totalling 4101 participants. The final analysis included data from 3534 of the participants, after removing incomplete datasets (no age (*n* = 36)), and participants outside the age range of 11 to 17 years (*n* = 531, of which *n* = 310 were from Nigeria). Of the 3534 participants, all had pre-theatre questionnaire data, 3420 had post-theatre questionnaire data, and 114 did not have matching post-theatre questionnaire data. The total number of answers for each item varied slightly (totals are added in tables for each question), as the questionnaire was administered on paper and some questions were skipped by participants. No consistent pattern was observed in the skipped questions.

Questionnaire demographics are presented in Table 1. The median age was 14 years. There was a higher proportion of female participants (68.9% vs 31.1% males), mostly driven by recruitment from Uganda since the Ugandan site had engaged several girls’ schools in the study.

**Table 1.** Participant details.

		Ghana	Malawi	Nigeria	S. Africa	Uganda	Zimbabwe	Combined
Participants	n	428	130	480	608	1421	467	3534
Age								
	Min-Max	11–17	11–17	11–17	11–17	11–17	12–17	11–17
	Median (Mean)	14 (13.96)	14 (13.97)	14 (13.90)	13 (12.78)	15 (14.64)	14 (13.41)	14 (13.95)
Sex								
male	% (n/total)	42.8 (183/428)	34.4 (44/128)	35.2 (169/480)	51.8 (313/604)	13.4 (189/1411)	42.1 (193/458)	31.1 (1091/3509)
female	% (n/total)	57.2 (245/428)	65.6 (84/128)	64.8 (311/480)	48.2 (291/604)	86.6 (1222/1411)	57.9 (265/458)	68.9 (2418/3509)
Schools/performances								
Schools	n	6	4	5	12	5	6	38
Performances to students	n	8	4	8	19	6	8	53
Performances including community	n	0	6	2	4	1	2	15

Overall, the educational theatre improved asthma knowledge and attitudes towards asthma for all relevant questions (Table 2) with a significant difference (*P* < 0.01) between before and after the performance.

**Table 2.** Understanding and attitudes of asthma before and after the performance.

Question			Ghana	Malawi	Nigeria	South Africa	Uganda	Zimbabwe	Combined
‘I feel I understand asthma’ ^a^	Before	% A/SA (n/total)	47.9 (205/428)	52.7 (68/129)	59.8 (287/480)	51.1 (310/607)	45.4 (637/1402)	47.8 (223/467)	49.2 (1730/3513)
		Median (25th, 75th perc.)	N (A,DA)	A (A,N)	A (A,N)	A (A,DA)	N (A,N)	N (A,DA)	N (A,N)
	After	% A/SA (n/total)	92.5 (394/426)	74.8 (92/123)	99.8 (479/480)	86.5 (499/577)	77.3 (1028/1330)	82.8 (386/466)	84.6 (2878/3402)
		Median (25th, 75th perc.)	A (SA,A)	A (SA,N)	SA (SA,SA)	A (SA,A)	A (A,A)	A (SA,A)	A (SA,A)
‘You can catch asthma from someone’ (not true)^a^	Before	% correct (n/total)	54.3 (232/427)	60.6 (77/127)	59.8 (287/480)	48.7 (296/608)	70.4 (990/1407)	46.5 (217/467)	59.7 (2099/3516)
	After	% correct (n/total)	89.5 (383/428)	72.0 (90/125)	82.7 (397/480)	63.4 (365/576)	82.2 (1093/1330)	72.8 (340/467)	78.3 (2668/3406)
‘Once you have asthma you have it for life’ (true)^a^	Before	% correct (n/total)	42.9 (183/427)	24.2 (31/128)	34.8 (167/480)	40.8 (248/608)	51.2 (718/1401)	46.5 (217/467)	44.5 (1564/3511)
	After	% correct (n/total)	70.7 (302/427)	39.8 (49/123)	52.7 (253/480)	53.6 (310/578)	61.4 (813/1325)	74.0 (345/466)	61.0 (2072/3399)
People with asthma can play sports (true)^a^	Before	% correct (n/total)	34.3 (146/426)	71.2 (89/125)	49.6 (238/480)	39.8 (242/608)	45.4 (637/1402)	39.8 (186/467)	43.8 (1538/3508)
	After	% correct (n/total)	96.3 (412/428)	69.8 (88/126)	99.4 (477/480)	80.6 (466/578)	95.9 (1210/1262)	83.3 (389/467)	91.1 (3042/3341)
Having asthma affects people emotionally (agree + strongly agree)^a^	Before	% A/SA (n/total)	52.9 (225/425)	37.2 (48/129)	58.5 (281/480)	51.7 (313/606)	55.8 (772/1384)	52.5 (245/467)	54.0 (1884/3491)
		Median (25th, 75th perc.)	A (A,DA)	N (A,DA)	A (A,DA)	A (A,DA)	A (A,DA)	A (A,DA)	A (A,DA)
	After	% A/SA (n/total)	80.3 (343/427)	48.4 (60/124)	99.6 (478/480)	78.0 (450/577)	84.2 (1119/1329)	73.9 (345/467)	82.1 (2795/3404)
		Median (25th, 75th perc.)	A (SA,A)	N (A,DA)	SA (SA,SA)	A (SA,A)	A (SA,A)	A (SA,N)	A (SA,A)
Support from friends and family is important ^a^	Before	% A/SA (n/total)	90.2 (385/427)	86.7 (111/128)	87.1 (418/480)	78.4 (476/607)	95.6 (1332/1394)	75.2 (351/467)	87.7 (3073/3503)
		Median (25th, 75th perc.)	SA (SA,A)	SA (SA,A)	A (SA,A)	A (SA,A)	SA (SA,A)	A (SA,A)	SA (SA,A)
	After	% A/SA (n/total)	96.5 (412/427)	91.3 (115/126)	99.8 (479/480)	84.9 (490/577)	98.5 (1300/1320)	86.1 (402/467)	94.1 (3198/3397)
		Median (25th, 75th perc.)	SA (SA,A)	A (SA,A)	SA (SA,SA)	SA (SA,A)	SA (SA,SA)	SA (SA,A)	SA (SA,A)
It is important to talk about asthma (agree + strongly agree)^a^	Before	% A/SA (n/total)	92.3 (395/428)	93.0 (119/128)	84.0 (403/480)	76.6 (466/608)	92.7 (1293/1395)	74.2 (346/466)	86.2 (3022/3505)
		Median (25th, 75th perc.)	A (SA,A)	SA (SA,A)	A (SA,A)	A (SA,A)	SA (SA,A)	A (SA,N)	A (SA,A)
	After	% A/SA (n/total)	94.6 (404/427)	88.8 (111/125)	99.6 (478/480)	86.5 (500/578)	96.1 (1278/1330)	79.0 (369/467)	92.2 (3140/3407)
		Median (25th, 75th perc.)	SA (SA,A)	SA (SA,A)	SA (SA,SA)	A (SA,A)	SA (SA,A)	A (SA,A)	SA (SA,A)
Controlling asthma may require medications (agree + strongly agree)^a^	Before	% A/SA (n/total)	63.3 (271/428)	87.3 (110/126)	82.7 (396/479)	66.0 (401/608)	71.6 (998/1393)	62.0 (289/466)	70.4 (2465/3500)
		Median (25th, 75th perc.)	A (A,N)	A (SA,A)	A (A,A)	A (SA,N)	A (SA,N)	A (A,N)	A (SA,N)
	After	% A/SA (n/total)	83.8 (356/425)	84.1 (106/126)	100.0 (480/480)	76.4 (441/577)	84.7 (1124/1327)	76.4 (357/467)	84.2 (2864/3402)
		Median (25th, 75th perc.)	A (SA,A)	A (SA,A)	SA (SA,SA)	A (SA,A)	A (SA,A)	A (SA,A)	A (SA,A)

^a^Wilcoxon-signed rank test between pre- and post-results: significant to reject the null hypothesis (*P* < 0.01). A, agree; DA, disagree; N, neutral; SA, strongly agree.

Asthma knowledge improved following the theatre performance. This is reflected in the answers to the question if the participants felt that they understood asthma, which rose from 49.2% (1730/3513) who agreed or strongly agreed prior to the theatre performance to 84.6% (2878/3402) after the performance. Ghana and Nigeria showed particularly large increases in affirmative responses from 47.9% to 92.5% and from 59.8% to 99.8%, respectively. An increase of correct answers to specific knowledge questions about asthma was also observed. Prior to the ‘In Control-Africa’ theatre, only 59.7% (2099/3516) of the total participants knew that asthma is not infectious, and cannot be transmitted from person to person. Post-theatre, correct answers increased significantly (*P* < 0.01) to 78.3% (2668/3406), with Ghana exhibiting the largest increase from 54.3% to 89.5%. The understanding that asthma is a chronic condition and that ‘once you have asthma, you have it for life’ also significantly increased (*P* < 0.01) post-theatre. Before the theatre, 44.5% (1564/3511) of respondents agreed or strongly agreed with the statement, which increased to 61.0% (2072/3399) after the theatre. This change was especially pronounced in Ghana and Zimbabwe with an increase from 42.9% to 70.7% and 46.5% to 74%, respectively. The increase in affirmative answers suggested that the theatre effectively communicated the chronic nature of asthma. Understanding that people with asthma can play sports rose significantly (*P* < 0.01), from 43.8% pre-theatre to 91.1% post-theatre.

Before the performance, about half of participants agreed or strongly agreed that there was an emotional effect for people with asthma (54%, 1884/3491), which significantly (*P* < 0.01) rose after the performance with 82.1% (2795/3404), and had the highest increase in Nigeria from 58.5% to 99.6%. Most participants agreed or strongly agreed that support from friends and family was important for people with asthma before the performance with 87.7% (3073/3503), which rose further to 94.1% (3198/3397) after the performance. Similarly, most participants agreed or strongly agreed that it was important to talk about asthma with 86.2% (3022/3505) prior to the performance, which rose further to 92.2% (3140/3407) after the performance. The perception that asthma may require medication was agreed or strongly agreed by 70.4% (2465/3500) of the participants before the performance, which increased to 84.2% (2864/3402) of participants after the performance.

### Feedback after the performance

Participants provided answers about how they perceived the performance after the educational theatre, which are summarized in Table 3.

**Table 3.** Feedback after the performance.

Question (Answer)		Ghana	Malawi	Nigeria	S. Africa	Uganda	Zimbabwe	Combined
Today I have learned about asthma	% A little/a lot (n/total)	99.3 (424/427)	98.5 (128/130)	100.0 (480/480)	96.2 (556/578)	99.2 (1307/1317)	94.2 (439/466)	98.1 (3334/3398)
	Median (25th, 75th percentile)	SA (SA,SA)	SA (SA,SA)	SA (SA,SA)	SA (SA,SA)	SA (SA,A)	SA (SA,SA)	SA (SA,SA)
Watching the theatre changed how I think or feel about asthma	% A/SA (n/total)	87.4 (374/428)	85.9 (110/128)	91.7 (440/480)	82.9 (479/578)	84.2 (1117/1327)	73.2 (342/467)	84.0 (2862/3408)
	Median (25th, 75th percentile)	A (SA,A)	SA (SA,A)	A (SA,A)	A (SA,A)	A (SA,A)	A (SA,N)	A (SA,A)
The theatre performance was fun	% A/SA (n/total)	93.9 (402/428)	88.5 (115/130)	96.0 (461/480)	92.7 (536/578)	92.5 (1232/1332)	75.2 (351/467)	90.7 (3097/3415)
	Median (25th, 75th percentile)	SA (SA,A)	SA (SA,A)	SA (SA,A)	SA (SA,A)	SA (SA,A)	A (SA,A)	SA (SA,A)
How did the performance make you feel?								
I want to help people with asthma	% Yes (n/total	62.4 (267/428)	41.9 (49/117)	55.6 (267/480)	73.0 (422/578)	59.1 (790/1337)	46.5 (217/467)	59.1 (2012/3407)
I understand people with asthma better	% Yes (n/total	46.7 (200/428)	17.9 (21/117)	44.0 (211/480)	59.5 (344/578)	30.7 (411/1337)	27.6 (129/467)	38.6 (1316/3407)
My opinion about asthma has not changed	% Yes (n/total	5.6 (24/428)	8.5 (10/117)	3.1 (15/480)	15.4 (89/578)	5.0 (67/1337)	6.9 (32/467)	7.0 (237/3407)
I am motivated to look after my own health better	% Yes (n/total	25.2 (108/428)	14.5 (17/117)	22.5 (108/480)	40.8 (236/578)	30.8 (412/1337)	14.8 (69/467)	27.9 (950/3407)
People should talk about health issues more	% Yes (n/total	39.0 (167/428)	17.1 (20/117)	45.4 (218/480)	50.0 (289/578)	51.9 (694/1337)	24.6 (115/467)	44.1 (1503/3407)

A, agree; DA, disagree; N, neutral; SA, strongly agree.

Almost all (98.1%, 3334/3398) participants agreed that they learned something (a little or a lot) about asthma that day. Furthermore, a large proportions of participants agreed or strongly agreed that the theatre influenced their thoughts and feelings about asthma (84.0%, 2862/3408) and agreed or strongly agreed that the performance was fun (90.1% (3097/3415).

When asked how the performance made them feel, 59.1% (2012/3407) said that they wanted to help people with asthma; 38.6% (1316/3407) said that the performance made them understand people with asthma better. Only 7.0% (237/3407) said that their opinion about asthma had not changed. For some participants, the performance influenced their thinking about health beyond asthma: 27.9% (950/3407) said that they are motivated to take better care of their own health, and 44.1% (1503/3407) agreed that people should talk more about health issues. Results by sex for knowledge, attitudes and feedback questions are presented in the supplement and show that male—female responses differ to some degree for all answers (between 0.7% and 7.2% points). These male—female differences are however far outweighed by changes in responses between before and after the performance. For example, before the performance 53.2% (578/1086) of male participants agreed with the statement ‘I feel I understand asthma’, compared to 47.5% (1141/2402) of female participants. After the performance the agreement with the statement rose to 88.0% (889/1010) for male participants and to 83.2% (1971/2368) for female participants. Yet, results of the sensitivity analysis show a significant influence of sex on all responses for pre-/post-knowledge and attitude questions.

## DISCUSSION

This study found the educational theatre about asthma, ‘In Control—Africa’, to be entertaining and educational. It succeeded in its aim of improving knowledge about asthma in urban African adolescents across several knowledge endpoints. The significant decline in the misconception that ‘asthma can be caught from someone else’ highlights that the theatre improved knowledge and dispelled myths that would contribute to stigma. This finding is particularly relevant in sub-Saharan Africa, where focus groups have shown a lack of understanding and common misconceptions about asthma (Naidoo *et al.* 2023a, 2023b). The substantial increase in awareness that asthma is lifelong and indicates that the theatre effectively conveyed essential health messages. By reinforcing the chronic nature of asthma, the theatre has the potential to improve adherence to treatment and long-term management practices. While the majority of participants were aware that asthma may require medication prior to the theatre, a further significant improvement in understanding the need for medication was observed, which is an important prerequisite towards improving health seeking behaviour in those with asthma symptoms. This is of particular importance in a population in which 80% of participants with asthma symptoms have not been diagnosed (Oyenuga *et al.* 2024). In addition, the performance shifted participants’ attitudes towards asthma, changed ‘how they think and feel about asthma’ and showed a high increase in the understanding that asthma has an emotional impact. An increased number of participants thought that people with asthma need to be supported by friends and family, and that it is important to talk about asthma. Health attitude changes through theatre have previously been reported for other health concerns by the WHO Europe report about ‘Evidence on the role of arts in improving health and well-being’, e.g. regarding nutrition, sexual health, as well as for a reduction in stigma towards dementia (WHO Regional Office for Europe 2019). The attitude changes from our ‘In Control—Africa’ theatre also translated into motivation to help people with asthma after the performance for the majority of participants. Following the theatre, participants reported feeling motivated to look after their own health better, and wanted to talk more about health issues. Responses to our questions differed significantly by sex, which reflects findings from several other studies, and is likely due to a combination of physical, as well as sociocultural and behavioural differences, such as gender roles, and symptom perception (Krenitsky-Korn 2011, Colombo *et al.* 2019, Boulet *et al.* 2022). A strength of our educational theatre is that it was adapted to fit each local context. The acceptability and positive perception of the theatre across countries and cultures suggests that this was successful for all local audiences. Our approach to making small changes to the original script at each site ensured that the health messages were not only received but understood within the cultural context of the participating communities, highlighting the importance of culturally sensitive approaches in global health initiatives.

Overall, this study adds to the growing body of evidence that creative, community-based approaches can play a pivotal role in public health education. The theatre format allowed us to link emotional engagement with increased knowledge, which likely contributed to the large improvements in knowledge and attitudes observed in this study (Mbizvo 2006). The interactive nature of the theatre, where participants discuss how to solve the situation for the main character, as well as ask questions after the performance, provided an opportunity for especially deep engagement and understanding (Bhaskar *et al.* 2012). Additionally, performing the theatre in a school setting potentially provided wider benefits through further information sharing and discussions among peers both within and outside their schools

However, the study had some limitations. First, our audience was sensitised to asthma research from earlier visits to the school by our team. It is possible that this may have improved some of the knowledge about asthma prior to the theatre. Second, the integration within the ACACIA study, e.g. combining school recruitment, meant that costs could not easily be calculated for the educational theatre alone. Future studies should include a cost-effectiveness analysis to inform scalability and integration into health systems. Third, we relied on self-reported data, which may introduce social desirability bias. The addition of a control arm with no theatre performances would have provided a more rigorous approach to assess effectiveness. Finally, the lack of a follow-up survey to assess if changes are maintained, limits understanding of whether knowledge and attitude changes are sustained over time, and if they may have led to behavioural changes. Future research should consider setting up a randomised controlled trial with a theatre and a control arm, should aim to validate the questionnaire, and assess long-term behavioural outcomes, as well as the potential integration into national school health programmes.

## CONCLUSION

The educational theatre ‘In Control—Africa’ significantly improved asthma knowledge and positively shifted attitudes among urban adolescents across six African countries. The combination of interactive theatre and culturally adapted scripts contributed to the theatre’s strong positive reception across all study sites. These findings provide compelling evidence that educational theatre is an effective, scalable tool for improving asthma knowledge and attitudes in diverse sub-Saharan African settings.

## Supplementary Material

daaf189_Supplementary_Data

## Data Availability

Data collected for the study, including deidentified individual participant data and a data dictionary defining each field in the set, will be made available. Data will be available without any restriction for 10 years after publication from the corresponding author, GM (g.mosler@qmul.ac.uk) upon request.
