# Supplementary material for: Using theatre to transform understanding and attitudes about asthma in African adolescents
Source: Health Promot Int. 2025 Nov 19;40(6):daaf189. doi: 10.1093/heapro/daaf189 (PMC12628735; doi:10.1093/heapro/daaf189)
Supplement: daaf189_Supplementary_Data [file daaf189_supplementary_data.docx]

Questionnaire results differentiated by male/female participants

Summary statistics for each question are presented by sex (male/female) below for 1,091 male participants out of 3,534 (30.9% of ), and 2,418 female participants (68.4%), as well as 25 participants who did not provide information on their sex (0.7%). Furthermore, a sensitivity analysis was undertaken for questions with pre- post- answers to determine if sex played a significant role in how participants answered. Sensitivity was tested using a logistic regression with the question as dependent variable and participant ID, pre/post, and sex as independent variables. A significant influence (p<0.005) of sex on outcomes is indicated by asterisk in the table below.

Table: Understanding and attitudes of asthma before and after the performance by sex

| **Question** |  |  | **male** | **female** | **all answers** |
| --- | --- | --- | --- | --- | --- |
| **"I feel I understand asthma" *** | Before | % A/SA (n/total) | 53.2 (578/1086) | 47.5 (1141/2402) | **49.2 (1730/3513)** |
|  |  | Median (25^th^, 75^th^ perc.) | A (A,N) | N (A,DA) | **N (A,N)** |
|  | After | % A/SA (n/total) | 88.0 (889/1010) | 83.2 (1971/2368) | **84.6 (2878/3402)** |
|  |  | Median (25th, 75th perc.) | A (SA,A) | A (SA,A) | **A (SA,A)** |
| **"You can catch asthma from someone"(not true)*** | Before | % correct (n/total) | 55.3 (602/1088) | 61.8 (1484/2403) | **59.7 (2099/3516)** |
|  | After | % correct (n/total) | 74.6 (753/1009) | 79.9 (1896/2373) | **78.3 (2668/3406)** |
| **"Once you have asthma you have it for life" (true)*** | Before | % correct (n/total) | 39.6 (431/1088) | 46.8 (1122/2398) | **44.5 (1564/3511)** |
|  | After | % correct (n/total) | 58.8 (593/1008) | 61.8 (1463/2367) | **61.0 (2072/3399)** |
| **People with asthma can play sports (true)*** | Before | % correct (n/total) | 47.1 (511/1085) | 42.5 (1019/2398) | **43.8 (1538/3508)** |
|  | After | % correct (n/total) | 87.6 (885/1010) | 92.7 (2139/2307) | **91.1 (3042/3341)** |
| **Having asthma affects people emotionally (agree + strongly agree)*** | Before | % A/SA (n/total) | 53.6 (579/1080) | 54.3 (1297/2388) | **54.0 (1884/3491)** |
|  |  | Median (25th, 75th perc.) | A (A, DA) | A (A,DA) | **A (A,DA)** |
|  | After | % A/SA (n/total) | 77.8 (785/1009) | 84.1 (1995/2371) | **82.1 (2795/3404)** |
|  |  | Median (25th, 75th perc.) | A (SA,A) | A (SA,A) | **A (SA,A)** |
| **Support from friends and family is important *** | Before | % A/SA (n/total) | 83.4 (906/1086) | 89.78 (2149/2394) | **87.7 (3073/3503)** |
|  |  | Median (25th, 75th perc.) | A (SA, A) | SA (SA,A) | **SA (SA,A)** |
|  | After | % A/SA (n/total) | 91.7 (927/1011) | 95.3 (2253/2363) | **94.1 (3198/3397)** |
|  |  | Median (25th, 75th perc.) | SA (SA, A) | SA (SA,A) | **SA (SA,A)** |
| **It is important to talk about asthma (agree + strongly agree)*** | Before | % A/SA (n/total) | 82.3 (895/1087) | 88.0 (2109/2396) | **86.2 (3022/3505)** |
|  |  | Median (25th, 75th perc.) | A (SA,A) | A (SA,A) | **A (SA,A)** |
|  | After | % A/SA (n/total) | 89.2 (902/1011) | 93.5 (2218/2372) | **92.2 (3140/3407)** |
|  |  | Median (25th, 75th perc.) | SA (SA, A) | SA (SA,A) | **SA (SA,A)** |
| **Controlling asthma may require medications (agree + strongly agree)*** | Before | % A/SA (n/total) | 71.0 (770/1084) | 70.3 (1682/2393) | **70.4 (2465/3500)** |
|  |  | Median (25th, 75th perc.) | A (SA,N) | A (SA,N) | **A (SA,N)** |
|  | After | % A/SA (n/total) | 81.7 (824/1009) | 85.3 (2021/2369) | **84.2 (2864/3402)** |
|  |  | Median (25th, 75th perc.) | A (SA,A) | A (SA,A) | **A (SA,A)** |

***Sex variable significant in regression at p < 0.005 level . Abbreviations SA=Strongly Agree, A=Agree, N=Neutral, DA=Disagree.**

Table: Feedback after the performance by sex

| **Question (Answer)** |  | **male** | **female** | **all answers** |
| --- | --- | --- | --- | --- |
| **Today I have learned about asthma** | % A little/a lot (n/total) | 96.9 (980/1011) | 98.6 (2331/2363) | **98.1 (3334/3398)** |
|  | Median (25^th^, 75^th^ percentile) | SA (SA,SA) | SA (SA,SA) | **SA (SA,SA)** |
| **Watching the theatre changed how I think or feel about asthma** | % A/SA (n/total) | 81.9 (829/1012) | 84.9 (2015/2372) | **84.0 (2862/3408)** |
|  | Median (25^th^, 75^th^ percentile) | A (SA,A) | A (SA,A) | **A (SA,A)** |
| **The theatre performance was fun** | % A/SA (n/total) | 89.8 (910/1013) | 91.2 (2169/2378) | **90.7 (3097/3415)** |
|  | Median (25^th^, 75^th^ percentile) | SA (SA,A) | SA (SA,A) | **SA (SA,A)** |
| **How did the performance make you feel?** | | | |  |
| I want to help people with asthma | % Yes (n/total) | 60.0 (606/1010) | 58.8 (1395/2374) | **59.1 (2012/3407)** |
| I understand people with asthma better | % Yes (n/total) | 42.1 (425/1010) | 37.2 (882/2374) | **38.6 (1316/3407)** |
| My opinion about asthma hasn’t changed | % Yes (n/total) | 9.3 (94/1010) | 5.8 (138/2374) | **7.0 (237/3407)** |
| I am motivated to look after my own health better | % Yes (n/total) | 26.9 (272/1010) | 28.4 (674/2374) | **27.9 (950/3407)** |
| People should talk about health issues more | % Yes (n/total) | 41.6 (420/1010) | 45.4 (1078/2374) | **44.1 (1503/3407)** |

**Abbreviations SA=Strongly Agree, A=Agree, N=Neutral.**
